# Operative management of penile strangulation caused by a rigid metallic ring: a case report

**DOI:** 10.1093/jscr/rjag781

**Published:** 2026-09-02

**Authors:** Naireen Asim, Moumn Abdalla, Siena Martin

**Affiliations:** Department of Urology, East Surrey Hospital, Canada Avenue, Redhill, Surrey, RH1 5RH, United Kingdom; Department of Urology, East Surrey Hospital, Canada Avenue, Redhill, Surrey, RH1 5RH, United Kingdom; St George's Medical School, City St George’s University of London, Blackshaw Road Tooting, London, SW17 0QT, United Kingdom

**Keywords:** penile strangulation, penile constriction injury, metallic ring, urological emergency, operative management, vascular compromise

## Abstract

Penile strangulation is a time-critical emergency in which delayed intervention risks vascular compromise and tissue injury. Management is heterogenous, varying with device characteristics, duration of incarceration, and severity of effects. We report a man in his seventies with penile strangulation caused by a metallic ring at the distal shaft, immediately proximal to the glans. Bedside removal (lubrication, topical 50% dextrose gel, and standard ring-cutter use) were unsuccessful owing to device rigidity. Given persistent entrapment, urinary difficulty, and evolving ischaemic changes, urgent operative removal was performed under general anaesthesia. Intraoperative findings included marked distal oedema, glans congestion, and early ischaemic changes, with resolution after division and removal. This case highlights the limitations of bedside intervention with rigid devices and the value of timely operative escalation. As a single case, these observations are intended to raise clinical awareness rather than establish generalizable escalation criteria, which would require larger, systematic series.

## Introduction

Penile constriction injuries represent an uncommon but well-recognized urological emergency, most frequently encountered following the use of constricting devices for sexual enhancement or autoerotic purposes [1–3]. The clinical challenge lies in the substantial variability between cases: management is influenced by the type of device, the duration of incarceration, and the degree of associated tissue injury. A range of removal techniques has been described, including lubrication, the string method, aspiration decompression, and orthopaedic or industrial cutting tools [2–5].

Metallic rings present a particular challenge because of their rigidity, which often renders conventional emergency department tools ineffective. While specialized cutting devices may be required, there is no widely accepted consensus on the optimal approach across differing clinical scenarios [2, 5].

We present a case of penile strangulation caused by a metallic ring that required operative removal following unsuccessful bedside attempts. This case illustrates the importance of early escalation in the presence of evolving vascular compromise and underscores that recognizing the limits of bedside intervention is a key component of safe management.

## Case report

An adult man in his seventies presented to the emergency department ~ 3 hours after placing a metallic ring around the distal penile shaft, immediately proximal to the glans, during sexual activity. He reported progressive swelling and pain of the glans penis, with an inability to pass urine since device application. He had initially been assessed at another facility and was referred for specialist urological evaluation. His past medical history was unremarkable, and he was taking no regular medications.

### Examination findings

On presentation, he was haemodynamically stable. The urethral meatus appeared healthy on external inspection, with no visible blood at the meatus and no other evidence of urethral injury on examination. The glans was swollen and engorged, with a small area of dark discolouration at the tip, first noted intraoperatively. There was marked distal oedema and tenderness, with early discolouration of the glans. The penile shaft distal to the ring appeared congested, with no evidence of overt skin necrosis or urethral bleeding. Distal sensation could not be reliably assessed at presentation owing to the degree of swelling.

### Initial bedside management

Initial bedside attempts at removal using lubrication were unsuccessful. A topical hyperosmolar agent (50% dextrose gel) was applied to the glans to reduce oedema and facilitate removal; no clinically significant reduction in swelling was observed. A standard emergency department ring cutter was subsequently employed but failed to divide the device owing to its thickness and rigidity. Given ongoing entrapment, urinary difficulty, and concern for evolving vascular compromise, a decision was made to proceed to emergency operative removal.

### Operative management

Under general anaesthesia, the patient was positioned supine, prepared, and draped in a sterile fashion. Intravenous co-amoxiclav was administered for infection prophylaxis in the context of tissue compromise. A rigid metal tongue depressor was inserted between the constricting ring and the underlying penile skin along its circumference to protect the soft tissues during cutting. The stainless-steel ring was then divided at two points using a diamond-tipped bone cutter, with the tongue depressor maintained as a protective barrier throughout and was removed in two segments.

**Figure 1:**
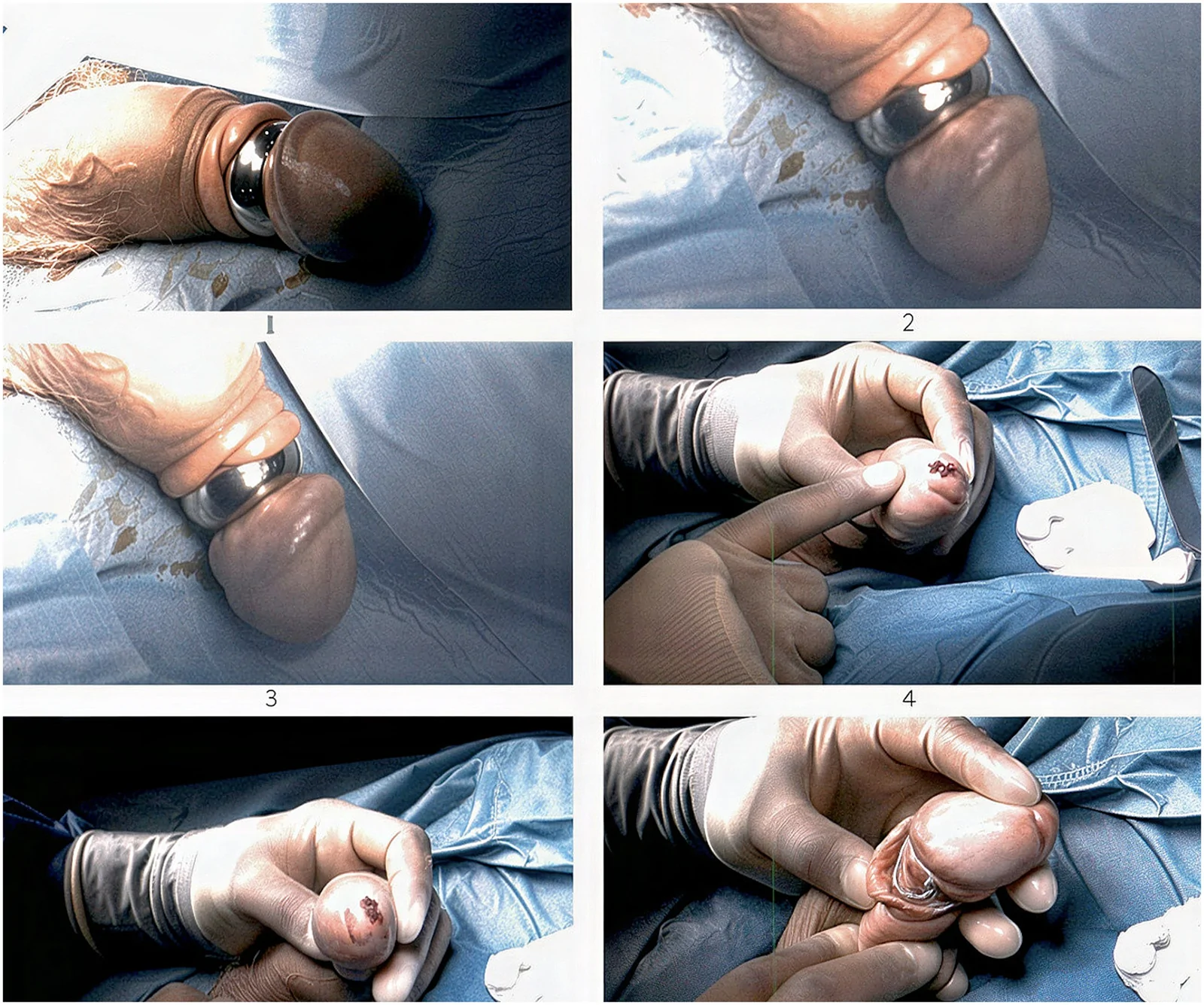
Intraoperative images demonstrating penile strangulation caused by a constricting metallic ring and the associated tissue changes. Composite image arranged from top left to bottom right, showing the constricting ring *in situ*, marked distal oedema and glans congestion, early discolouration consistent with evolving ischaemic change, and the post-removal appearance.

Intraoperative findings included significant distal oedema, congestion of the glans, and a small area of discolouration at the glans tip (Fig. 1). The tissue beneath the ring appeared inflamed and friable, consistent with early ischaemic change. Following removal of the device, there was resolution of glans congestion, indicating restoration of perfusion. The area was irrigated with povidone-iodine, topical antibiotic ointment was applied, and the foreskin was repositioned over the glans.

### Postoperative course

Postoperatively, the patient was monitored on the ward. Routine blood tests on the first postoperative day were unremarkable, and he was able to void spontaneously without difficulty. At review three days after surgery, mild swelling of the foreskin was noted, with no evidence of infection, dehiscence, or further tissue loss; the patient remained comfortable and continued to void normally. He was discharged towards the end of that week with a further seven-day course of oral co-amoxiclav and was counselled regarding avoidance of constricting devices during sexual activity.

The long-term outcomes assessed in the week were as follows: urinary function: normal voiding, no dysuria, no abnormality of stream, penile/glans sensation: subjectively normal, wound healing: complete healing with mild residual induration, urethral complications: none identified, and delayed tissue necrosis: none observed. The patient was safety-netted to arrange prompt urology review if any new changes occurred.

## Discussion

Penile constriction injuries encompass a spectrum of pathology in which clinical severity is shaped by both the duration of incarceration and the physical characteristics of the constricting device. Several grading systems have been proposed to standardize injury description, most notably that of Bhat *et al*. [1], which stratifies cases according to distal oedema, skin involvement, urethral injury, and tissue necrosis. While useful for comparison across reports, such classifications offer limited guidance for real-time management decisions.

The literature on penile strangulation tends to focus on the technique ultimately employed for device removal, with comparatively less attention given to the sequence of bedside attempts or the criteria used to determine when escalation is warranted [3, 5, 6]. As a result, the threshold at which continued bedside attempts become ineffective or potentially harmful remains poorly defined. This gap is clinically significant given the underlying pathophysiology: progressive venous and lymphatic obstruction exacerbates distal oedema, which in turn increases the risk of arterial compromise as constriction persists [1, 4]. In this setting, prolonged bedside manoeuvres may compound tissue injury while delaying definitive intervention.

In the present case, these considerations supported early escalation to operative management after two bedside modalities combined with attempted ring-cutter division, failed to achieve removal. Surgical removal allowed controlled division of the device with tissue protection using a tongue depressor, minimizing iatrogenic injury. Prompt restoration of perfusion, evidenced by resolution of glans congestion immediately after removal, is consistent with previous reports describing reversibility of vascular compromise when timely decompression is achieved [4, 5]. Notably, early ischaemic change was already evident at the time of intervention despite a relatively short incarceration period of approximately three hours, raising the possibility that rigid metallic rings may shorten the window before clinically apparent tissue injury develops, though this cannot be established from a single case.

Functional and longer-term outcomes after penile strangulation remain sparsely reported. Campbell et al. highlight that post-extrication follow-up and documentation of sequelae—including erectile function and urethral outcomes—are inconsistently captured in the literature, limiting understanding of the true morbidity associated with these injuries [6]. In the case described here, the presence of inflamed and friable tissue beneath the ring, together with preoperative urinary retention, indicates that even a brief period of entrapment can produce clinically significant local effects, reinforcing the need for structured follow-up that extends beyond confirmation of successful device removal.

## Conclusion

This case demonstrates that rigid metallic constriction rings may resist standard bedside removal techniques and can be associated with early ischaemic change even after a relatively short duration of incarceration. Early escalation to operative removal, with attention to tissue protection during device division, allowed prompt restoration of perfusion and an uncomplicated short-term recovery in this patient. As findings from a single case, these observations are offered to raise clinical awareness rather than to establish general management criteria. Larger, prospectively collected case series with standardized, structured long-term follow-up are needed to define objective criteria for escalation and to clarify the true incidence of functional sequelae following penile strangulation.
